# Multiomics of Aging and Aging-Related Diseases

**DOI:** 10.3390/ijms252413671

**Published:** 2024-12-21

**Authors:** Olga I. Kiseleva, Viktoriia A. Arzumanian, Yuriy A. Ikhalaynen, Ilya Y. Kurbatov, Polina A. Kryukova, Ekaterina V. Poverennaya

**Affiliations:** 1 Institute of Biomedical Chemistry, Pogodinskaya Street, 10/8, 119121 Moscow, Russia; arzumanian.victoria@gmail.com (V.A.A.); ikh.ya@yandex.ru (Y.A.I.); kurbatild@gmail.com (I.Y.K.); polinakpa@gmail.com (P.A.K.); k.poverennaya@gmail.com (E.V.P.); 2 Chemistry Department, Lomonosov Moscow State University, 119991 Moscow, Russia

**Keywords:** aging, multiomics, aging-related diseases

## Abstract

Despite their astonishing biological diversity, surprisingly few shared traits connect all or nearly all living organisms. Aging, i.e., the progressive and irreversible decline in the function of multiple cells and tissues, is one of these fundamental features of all organisms, ranging from single-cell creatures to complex animals, alongside variability, adaptation, growth, healing, reproducibility, mobility, and, finally, death. Age is a key determinant for many pathologies, shaping the risks of incidence, severity, and treatment outcomes for cancer, neurodegeneration, heart failure, sarcopenia, atherosclerosis, osteoporosis, and many other diseases. In this review, we aim to systematically investigate the age-related features of the development of several diseases through the lens of multiomics: from genome instability and somatic mutations to pathway alterations and dysregulated metabolism.

## 1. Introduction

Advances in modern healthcare have significantly improved human well-being and extended life expectancy [1]. Globally, life expectancy at birth has increased substantially from 1990 to 2017, rising by approximately 7.4 years, from 65.6 years to 73.0 years [2]. While historical data show a steady increase in life expectancy, there is ongoing debate about whether there is an upper limit to the human lifespan. Some studies suggest a potential natural "ceiling", particularly after age 100, while others argue for the possibility of winning over aging.

Aging is a progressive and irreversible pathophysiological decline in cellular and tissue functions accompanied by a marked increase in the risk of various age-related diseases (including cardiovascular, neurodegenerative, oncological, metabolic, musculoskeletal, and several other disorders) becoming the primary contributors to disability and mortality in the elderly [3]. Over 40% of individuals aged 40 and older experience cardiovascular disease (CVD), with prevalence rising to 80% by the age of 80 [4]. In the case of neurodegenerative disorders, which profoundly impact both functional capacity and quality of life, onset typically occurs in middle age (50–60 years), as observed in conditions such as Alzheimer’s disease. Age over 40 also constitutes a significant risk factor for the development of type 2 diabetes, with approximately 24% of individuals over 70 affected by this condition [5]. Of the 150 thousand deaths per day across the globe, about two-thirds are caused by age-related reasons [6].

To decelerate aging, it seems essential to establish an ideal molecular profile of healthy aging, separating the beneficial markers from the noise—essentially, distinguishing characteristics of healthy aging from disease-associated traits. Extreme longevity in individuals might serve as a positive example of healthy aging; however, their health metrics often diverge significantly from general population references, introducing confounding factors in defining a molecular portrait of aging [7].

Advanced nucleic acid sequencing technologies provide researchers with sensitive, comprehensive data on the genome and transcriptome of aging [8,9]. Methylome profiling, known for its stable correlation with chronological age, allows for age predictions with an accuracy of up to two years [10]. Proteomic data, while less sensitive than nucleic data, account for translational regulation and post-translational modifications associated with senescence [11,12]. Metabolomic data, though more variable, allow for a detailed exploration of gene expression changes at a phenotypic level across a diversity of aging processes [7].

Over the past few decades, high-throughput technologies have generated a substantial body of aging-associated research across various levels of genetic information transmission, from the epigenome to the microbiome. However, most of these studies are limited in scope, often focusing on only one or two omics levels. Through extensive profiling, dozens to hundreds of potential markers of aging processes have been identified at each molecular level [13]. However, findings at one omics level are generally not transferable to another. For instance, the Spearman correlation coefficient between transcripts and their corresponding proteins fluctuates around 0.4 to 0.6 [14].

The integration of multiomics data is crucial for aging research as it offers a comprehensive understanding of the complex biological processes involved in aging and facilitates the development of novel anti-aging interventions [15]. Analyzing and interpreting data separately overlooks the correlations and biological interactions between different omics layers. By combining data across various omics levels, researchers can obtain a multidimensional perspective on the aging process, enabling the identification of underlying mechanisms and interactions driving aging [16].

The hope for constructing such a profile within a highly heterogeneous sample of older individuals primarily rests on multiomics methods. Multiomics approaches offer a robust framework for investigating the complex links between aging and pathology by enabling the examination of compensatory changes and clarifying where causative mechanisms transition into adaptive responses [17]. This review aims to compile a digest of multiomics research on human aging within the context of age-associated diseases.

## 2. With Age Comes Diseases, but Sometimes Age Comes Alone

Although “healthy aging” serves as a cornerstone term for gerontology, there is no universally agreed-upon definition for it. The closest attempt to formalize this term has been made by the World Health Organization (WHO), which defined healthy aging as “the process of developing and maintaining the functional ability that enables wellbeing in older age” [18]. Functional ability, in turn, is described as “having the mental and physical capabilities to enable people to be and do what they value”. A functionally capable individual can meet their basic needs, can learn, grow, and make decisions, is mobile, can establish and maintain relationships, and, overall, is an active member of society [19].

A challenge in the field of markers of old age is the need to find differences between aging and disease. Epidemiological studies involve many people with diseases and disabilities, and more of them as they age. Therefore, it is difficult to understand which biomarker changes are associated with normal aging and which are associated with disease. Some studies are able to take into account the presence of a disease in each individual participant, so in these studies, the markers identified may be considered to be unrelated to the disease. However, strict control over the health status of the volunteers in the selection process may lead to results that differ from the traditional picture.

For example, in Toshiko Tanaka’s study emphasizing the health of the described sample, the proteomic profiling of 240 healthy men and women aged 22–93 years revealed 188 proteins positively correlated with age and 22 proteins were negatively correlated. Some of the classical biomarkers of aging such as IL6, TNFα, and IGF-1 were not among the proteins significantly associated with age, but this result may be explained by the exceptional health status of the individuals in this study. All volunteers were very healthy, according to the strict criteria originally developed for inclusion in the BLSA [20].

As a second point, to better understand the aging process it is necessary to investigate not only the differences between young and old people but also between healthy and sick elderly people. In a study conducted by Alejandro Santos-Lozano [21], the blood plasma proteomes of nine long-lived people and nine people who died of age-related diseases were compared. The results revealed 49 proteins whose expression differed significantly between the two groups. These changes in the proteome indicate that healthy long-livers have a more active expression of proteins involved in the formation of new blood vessels and the maintenance of cellular integrity. At the same time, the expression of proteins associated with the development of cardiovascular diseases is reduced in them [21].

A significant obstacle to a deeper understanding of the aging process is the focus of research on individual omics layers. Many of the earlier works focus on detailed studies of proteomic, metabolomic, or other layers [13,16]. However, the number of comprehensive studies that utilize a multiomics approach and pay due attention to the health status of the study group still needs to be increased.

One example of large-scale multiomic profiling is found in the research conducted by M. Snyder’s laboratory. In this study, detailed health data was analyzed from 106 participants aged 29 to 75. Various indicators, including gene transcripts, protein levels, metabolites, cytokines, microbial communities, and clinical laboratory measurements, were examined to determine how they change with age. A total of 10,343 genes, 306 proteins in plasma, 722 metabolites, 62 cytokines, and 6909 microbial species (at the 16S rRNA level) as well as 51 clinical parameters were analyzed. For each indicator, a Spearman correlation analysis was performed concerning the mean age of participants. After adjusting for body mass index (BMI) and gender, 184 molecules across different ‘omics’ were identified that demonstrated distinct trends and levels of association with age [17].

In addition to the difficulties associated with the construction of age models due to various diseases that can significantly affect the multiomics profiles studied, other factors must be considered. Sex, ethnic, and racial differences can substantially affect the interpretation of results [22]. In large cohort studies, these differences are necessarily taken into account [23].

In addition, the influence of socioeconomic, environmental, and lifestyle factors on the aging process cannot be underestimated [24]. However, these factors are difficult to assess in broad multivariate studies. One of the few studies on the relationship between sociocultural factors and biomarkers (DNA methylation level) of aging shows that the level of education is associated with accelerated biological aging [24].

Although comprehensive studies of multiomics markers of aging have yet to be widespread, the study of this process at various levels is actively pursued, which has led to the accumulation of a significant amount of data. HALL (Human Ageing and Longevity) is a versatile web-based resource that opens new horizons for the study of human physiology and pathology across the lifespan. Created under the auspices of the Aging Biomarkers Consortium (ABC), HALL is a unique and valuable tool that fills a significant gap in research related to aging and longevity. All data in HALL are manually collected from the literature and extracted from existing databases, making it a comprehensive and reliable source of multivariate datasets [25]. There are other databases containing information on aging (Table 1), such as The Digital Ageing Atlas [26], AgingBank [27], or AgeAnnoMO [28]. However, the data libraries in these databases have either not been updated for a long time or contain a large amount of information about other organisms, making them difficult to use in analyzing healthy human aging.

There is an active research effort to develop a biological clock based on various omics data such as epigenomics, transcriptomics, proteomics, metabolomics, and circulating blood biomarkers [29]. By combining these methods using artificial intelligence, researchers created a biological clock for each organ. Such a clock can predict the development of age-related diseases with high accuracy, which opens up new perspectives in the field of health care [30].

**Table 1:** Omics databases dedicated to human aging.

Database	Omics Data	Available Organisms	Last Update	Ref.
HALL	phenomics, genomics, transcriptomics, proteomics, metabolomics, and microbiomics	human	2023	[25]
Aging Atlas	genomics, epigenomics, transcriptomics, proteomics, metabolomics, and pharmacogenomics data	human, mouse	2024	[31]
AgeAnnoMO	genomics, proteomics, metabolomics, mitochondrial genomics, and microbiomics	50 representative species	2024	[28]
AgingBank	genomics, transcriptomics, and epigenomics	50 model organisms, including human, mice, worms etc.	2022	[27]
MetaboAge DB	metabolomics	human	2020	[32]
Human Ageing Genomic Resources (HAGR)	genomics	human	2018	[33]

## 3. Cardiovascular Disease

According to the WHO’s data, cardiovascular diseases (CVDs) remain the main cause of death around the globe. The risks of CVD manifestation increase with age [34]. By prognosis, in some countries, by 2030 up to 20% of the population will be over 65 years old, and 40% of this cohort might die from CVDs [35]. This hazard is particularly relevant for developing countries with poor disease diagnosis [36].

The leading age-dependent diseases of the cardiovascular system are coronary heart disease (CHD), hypertension, heart failure (HF), and stroke [37]. These conditions mainly progress under the influence of atherosclerosis, the buildup of plaques in the arteries.

They might look dissimilar in clinical expression, but the underlying mechanisms are believed to be in accordance with the common route of aging: starting with telomere shortening and further epigenetic changes that lead to the accumulation of dysfunctional molecules, impaired DNA restoration, and total cell maladjustment. The inevitable change concerns all types of the cardiovascular system’s cells, with each one’s transformation bringing its contribution to the shaping of different age-related CVDs [38].

By now, due to the high interest in the diagnosis, the markers of cardiovascular aging-associated diseases have indeed been characterized on various layers [39,40,41]. However, it seems that their comprehensive integration utilizing multiomics studies can provide novel insights for our understanding. Quite naturally, there are not so many works in that field.

For example, it was shown that epigenetic age, which is calculated by the changes in DNA methylation (commonly in the areas of CpG islands), rather than chronological age, can be a more reliable marker for atherosclerosis prognosis [42]. The difference between these two types of age measures is called epigenetic age acceleration (EAA), and it was demonstrated that healthy middle-aged individuals with subclinical atherosclerosis (SA) exhibit this difference [43]. Implementing the statistical model-based causal mediation analysis for proteomic and transcriptomic data, it was shown that there is a link between subclinical atherosclerosis and EAA. It mainly concerns pro-inflammatory pathways (Th1/2 activation pathways, the STAT3 pathway, interleukin (IL)-10 signaling, and Toll-like receptor signaling) and genes (*IL1B*, *OSM*, *TLR5*, and *CD14*). This finding is no surprise, as it is in accordance with the concept that chronic inflammation is an inevitable mark of aging and, as well, the promotion of CVDs [44].

Among all CVDs, heart attack and stroke are the most dangerous and lethal. The investigators suggested that combining evidence from various ‘omics’ could help dissect the genetic architecture of heart failure [45]. They studied the participants from the Framingham Heart Study with a mean age of 55 years. By the integration of GWAS, gene expression, and DNA methylation data from the patients’ blood samples, the set of genes was suggested to be associated with the development of this disease’s traits, such as left ventricular systolic and diastolic function, remodeling, and prevalent and incident heart failure with reduced or preserved ejection fraction. Some of the top discovered genes are associated with the cardiomyocyte structure or actin cytoskeleton function (*MTSS1*, *MYCL*, *ANK1*, *RAB11FIP3*), intercellular communication and junctions (*ITGA9*, *TSPAN16*), matrix reshapement (*MMP20*), and cytokine signaling (*C5*). Some others have been previously reported to be HF (*MMP20*, *C5* [46]) and hypertension (*HPCAL1* [47]) markers or have been detected to have an influence on normal heart function (*NUP210*).

Another study [48], which aimed to dissect HF prediction markers, combined the aptamer-based proteomic profiling of plasma derived from 654 patients 30 days after MI with single-cell transcriptomics (from both mouse models of MI and HF and human) for validation. As a result, out of more than 200 differently expressed plasma proteins, 6 top protein candidates were associated with the development of post-myocardial infarction heart failure. These were the two well-known biomarkers of HF, MI, and heart stress NT-proBNP/BNP-32 [49] and troponin T [50], as well as promising markers angiopoietin-2 [51] and thrombospondin-2 [52], and the newly outlined latent transforming growth factor beta binding protein 4 and follistatin-related protein 3 [53]. However, the rest of the proteins, which were ranked lower, might be useful for future investigation and need mining.

The multiomics approach can be implemented for not only diagnosis but also for the improvement of current treatment and for finding new therapeutic targets. Though the aim of the research was the investigation of pathways associated with high lethality among patients with HF (these were the PI3K/Akt pathway, the MAPK pathway, the Ras signaling pathway, and epidermal growth factor receptor tyrosine kinase inhibitor resistance), the data from various layers (genetic, transcriptomic and proteomic) also highlighted some powerful disease markers (see in Table 2), among which are the abovementioned NT-proBNP and troponin T [54].

Another research group implemented the set of omics (genetics, epigenetics, transcriptomics, and metabolomics), aiming to find a certain link between some known CVD marker metabolites, among which was circulating short-chain dicarboxylacylcarnitine (SCDA), and pathological pathways [55]. Their findings suggest an association between SCDA and the regulatory genes of endoplasmic reticulum stress. Indeed, stressed ER can lead to improper protein synthesis and folding, lipid biosynthesis, and the impairment of homeostasis, and its contribution to the development of various CVDs is supported for hypertension, ischemic heart disease, heart failure, aortic aneurysm, and diabetic cardiomyopathy [56]. The researchers additionally proved this model by the exposure of cell cultures to the levels of fatty acids found comparable to those of individuals with cardiometabolic disease. The cells showed the accumulation of SCDA metabolites and endoplasmic reticulum stress markers.

Finally, it is important to explore normal heart aging. One study compared the proteomic and transcriptomic profiles of left ventricle samples that were obtained from healthy human hearts of young (<30 years old) and aged (>50 years old) patients [57]. The hallmarks of disease-independent “heart aging” are related to the change in mechanical properties of the heart. For example, these include the increase of collagen and fibrillin ratio and proteoglycans (decorin and lumican), which make collagen fibrils thicker. In addition, the amount of cytokines (such as IL-1a, IFN-y, angiopoietin-2 (which was also discovered by another group [48]), Dkk-1, Emmprin, SDF-1α, and SerpinE1) is elevated. The results of proteomapping suggested enrichment in the NF-KB, MAPK, and HIF-1α signaling pathways.

The multiomics approach uncovered the shared pathways for CVD development that are logically related to inflammation and stress. However, the question is whether they are distinct for CVDs, or go together with standard aging processes. With the characteristic features of the cardiovascular system, unique markers, such as NT-proBNP and troponin T, normally expose themselves. On the other hand, there is a trend when various combinations of omics and objects may discover additional markers. The transomic approach normally generates large amounts of data, which might need additional mining for the dissection of new and prominent hallmarks.

**Table 2:** Summary of multiomics research discoveries on cardiovascular diseases.

#	Omics layers					Bullet Points	Ref.
	DNA/Methylated DNA	RNA	Proteins	Metabolites and Lipids	Microbiota		
1	-	✓	✓	-	-	Top markers associated with SA on transcriptomic layer are *IGHA2*, *ORM1*, *MCEMP1*, *F5*, *MFHAS1*, *GPR55*, *SCART1*, *LPCAT3*, *VCAN*, *LRRN3*, on proteomic level: PIGR, PRG4, APOB, IGHA2, SYF1, PI16, Q5EFE5, HEP2, APOC, and FA10; Using mediation analysis, the *IL1B*, *OSM*, *TLR5*, and *CD14* genes were found to mediate the association between SA and EAA; The major discovered pathways are inflammation-related: Th1/2 activation pathways, the STAT3 pathway, interleukin (IL)-10 signaling, and Toll-like receptor signaling.	[43]
2	✓	-	-	-	-	Major genomic contributors to the development of HF are *IANK1*, *ANKRD13D*, *C5*, *HPCAL1*, *ITGA9*, *MMP20*, *MTSS1*, *NUP210*, *PTTG1IP*, *RAB11FIP3*, *TRIM69*, *TSPAN16*, *ZFP3*, and *ZNF146*; These genes operate cardiomyocyte structure and function, actin cytoskeleton function, cytokine signaling, intercellular communication and junctions, matrix reshapement; The authors also point out the possible limitation in generalization due to the fact that the analysis was performed from the samples of individuals of mainly white European ancestry.	[45]
3	-	✓	✓	-	-	Most of the predictors for post-MI HF are matricellular secreted proteins; Six top markers were found in the study: widely known NT-proBNP/BNP-32 and *TNNT2*, as well as newly discovered *ANGPT2*, *THBS2*, *LTBP4*, and *FSTL3*.	[48]
4	✓	✓	✓	-	-	The investigators collected 4 panels of markers for evaluation (phenotypic, comprising 54 traits; protein, transcriptomic, and genomic) for the prediction of HF-related mortality; In the clinical panel, the history of renal disease, renal failure, and edema exhibited most importance. Top protein (WFDC2, TRAIL-2R, FGF23RUML, ADM, LILRB4, NT-proBNP, troponin T, pro-ENK, and hemoglobin), transcriptome (*TRAJ16, TRAJ13, TRAJ31, SLAMF6*, and *TRAJ20*), and genome (rs2894240_G, rs11629005_C, *LOC105376544*, and rs315799_C, *LINC00840*) markers are listed; PI3K/Akt pathway, the MAPK pathway, the Ras signaling pathway, and epidermal growth factor receptor tyrosine kinase inhibitor resistance were shown to contribute to HF progression. Altogether, these pathways contribute to the decreased activation of the cardioprotective ERBB2 receptor.	[54]
5	✓	✓	-	✓	-	The mean age of participants (the members of the CATHGEN study) was 57.6 y.o. in the discovery group and 62.2 in the validation one. The clinical panel included lifestyle traits (smoking), correlated diseases and family history. Race-stratification was also implied; Among the set of metabolites (SCDA, LCDA, and MCA), the genetic loci associated with SCDA were strong enough for CVD prediction; The key discovered genes (*USP3*, *HERC1*, *STIM1*, *SEL1L*, *FBXO25*, *SUGT1*, *BRSK2*, and *HOOK2*) are associated with the regulation of endoplasmic reticulum stress.	[55]
6	-	✓	✓	-	-	The discovered features of disease-independent heart tissue ageing are connected with the changes in its mechanistic properties (the decrease of fibronectin and glycoprotein and elevation of collagen and fibrilin) and the increase in cytokines such as resistin, pentraxin 3, kallikrein 3, SerpinE, IL-1a, IFN-y, angiopoietin-2, BDNF, etc. On a transcriptomic level, the elevation of *NPPB* was detected.	[57]

## 4. Neurodegenerative Diseases

Aging is thought to be a major risk factor for neurodegenerative diseases (NDs) which come from decreases of some of the neuron populations (weak ones). Nerve cell degeneration is characterized by various molecular-level processes, including DNA damage, the formation of abnormal protein conformers, hormone level alteration, etc. Due to the complexity of processes related to the formation of ND pathology, there is still no clear way to describe origins even for common NDs, such as Alzheimer’s disease (AD) and Parkinson’s disease (PD), and their connections with aging. The multiomics approach is a promising tool for evidence search.

AD is one of the most frequently diagnosed NDs because of the capabilities of preclinical stage detection and its actual degree of incidence among populations. The molecular basis of AD is connected with specific polypeptide-based amyloid formation and tau-protein hyperphosphorylation, which leads to neurofibrillary tangle formation. It is also associated mostly with the elderly part of the population; however, young-onset disease also occurs [58,59].

### 4.1. Lipidome Changes Based on Insights from the Overall Multiomics AD Profile

The impact of AD pathogenesis on neural cells’ functions is proven to be strongly associated with membrane composition changes: metabolism dysregulation of various lipid classes, including cholesterol and its metabolites, sphingolipids, and gangliosides, leads to changes in lipid rafts’ structure, which plays a key role in the aggregation of amyloid peptides, which are main neurotoxic species involved in AD [60]. The development of lipidomic tools (both instrumental and data processing-related) allows researchers to investigate new lipid markers of AD and its progression using a one-omic-centered approach [61,62]. Nonetheless, unfortunately, the absence of other molecular-level data embarrasses the actual biochemical interpretation of discovered markers and the formation of diagnostic panes.

An opposite approach for lipid marker discovery is to transition from the results of higher levels of omics analysis to the pool of target metabolites. An interesting implementation of this strategy was presented by Monica Emili Garcia-Segura et. all [63]. Authors combined several transcriptomics and proteomics datasets obtained during the analysis of AD-related transgenic (with the presence of mutations in genes involved in amyloid and tau aggregation and processing) mouse models and GWAS AD-related orthologs through the implementation of DE (differential expression) analysis steps and Gene Ontology (GO) mapping. As a result of GO analysis, DEGs, implicated in mitochondrial and energy metabolism, lipid metabolism, and oxidative stress, were identified and involved in further metabolic subnetwork extraction with a focus on lipid biosynthesis and cholesterol metabolism. The resulting lipid signatures likely to be connected with AD included 133 lipid species with two orders of connectivity with selected metabolic pathways, and validation proceeded on the mice dataset, followed by a further MWAS concordance test on two human datasets. Significant coherence among the mice and human metabolomic datasets was found, even with different methods (HPLC-MS and NMR) used, with the dysregulation of cholesterol and fatty acids metabolism and glycerophospholipids biosynthesis.

A ceramide pathway-based multiomics study was conducted by Priyanka Baloni et al. [64]. Postmortem brain transcriptome data, neuroimaging, in-silico metabolic flux, and lipidomics/metabolomics plasma profiles were analyzed to identify potential treatment targets. The transcriptomic analysis of brain data showed the significant dysregulation of 20 out of 35 genes involved in the SM/Cer pathway in the case of AD patients, including sphingomyelin synthases and ceramide kinase, which were significantly dysregulated across multiple brain regions in the studied cohort. Pathway flux analysis confirmed ceramide kinase catalyzed reaction differences among control and patient groups. As metabolite ratios were taken into account during possible marker compounds’ discovery, SM (d34:1)/SM (d43:1) showed associations with AD and cognition loss. Additionally, sphingosine-1-phosphate metabolism was identified as a key pathway involved in AD progression, so authors performed APP/PS1 mice tests with fingolimod (an S1P receptor modulator) to evaluate the efficiency of the discovered target modulation in AD treatment. Fingolimod improved cognitive function and reversed synaptic plasticity deficits, which proves that sphingolipid metabolism is a possible therapeutic target for AD.

An interesting approach to process metabolome and proteome data is described in a paper by Alicia Gómez-Pascual et al. [65]: the authors investigated the progression of cognitive dysfunction through the analysis of AD-diagnosed patients, patients with mild cognitive impairment (MCI), and subjects with normal cognition. A key feature of the implemented study design is the usage of multiclass ML-based (machine learning) models for the discovery of proteins and metabolites with a significant impact on over-group changes and MCI development. The authors proceeded with omics data separately during the multiclass models’ construction and evaluation and then combined them during MCI development. Only the metabolome level markers showed their impact in both classification and conversion models, including oleamide, an endocannabinoid, which is characterized by its implication on neural functions. As validation, in vitro experiments were conducted and found that oleamide was secreted by microglia in extracellular vesicles; in particular, AD cases’ concentrations were much higher than those in non-AD groups, which correlates with previous studies on fatty amide metabolism’s connection with AD [66]. Along with other proteins, phospholipase A2 is elevated in MCI individuals, which correlates with membrane-connected disease development as a part of aging-related processes, as mentioned before.

### 4.2. Exosome AD-Related Multiomics Studies

As discovered in the work above [65], processes in microglial cells are strongly connected with AD progression [67], which also correlates with the pivotal role of microglia during aging [68,69] and the regulation of the impact of detrimental processes. Another microglia-related study was presented by Whitaker Cohn et al. [70]. Researchers focused on microglia extracellular vesicles (EVs) in AD/non-AD brain tissue samples. Proteomics essay revealed the upregulation of disease-associated microglia markers and several synaptic functions and neural degradation-related proteins. miRNA profiling discovered an upregulation of miRNAs associated with immune and cellular dilapidation pathways. Lipidome-based markers were associated with the dysregulation of defects in acyl-chain remodeling, lysosomal dysfunction, and cholesterol upregulation.

The integration of publicly available proteomics and transcriptomics brain tissue-based datasets with newly assembled stem cell-derived EV proteomics data was performed by Morteza Abyadeh et al. [71]. The EV data were separated by the sizes of the extracted vesicle populations and the differential expression in the external datasets was compared with every vesicular group. Larger EVs showed the most potential in terms of their protein content. This subpopulation had the highest number of mitochondrial-associated proteins related to energy metabolism and inflammation. Inflammatory pathways were upregulated in AD brains but not in the EV subpopulations. The authors also highlight several EV-related proteins as regulators of mitochondrial processes, including energy metabolism.

In both gerontology and aging-related (especially neurodegenerative) disease studies, membrane-associated processes are one of the most promising fields for the accumulation of molecular basis knowledge. The usage of proper multiomics techniques could highlight more important details. In some situations, it also serves as orthogonal evidence, which is essential in AD cases. On the other hand, there are several sources of data, including tissue samples, biological liquids, and EVs, some that need to be better studied. Studies on EVs could give more insights into the actual signaling basis of AD and provide information for the discovery of potential therapeutic targets. As a conclusion, we provide a summary table about AD-related multiomics studies, including some unmentioned above (Table 3).

**Table 3:** Summary of multiomics research discoveries on Alzheimer disease.

#	Omics Layers				Bullet Points	Ref.
	DNA/Methylated DNA	RNA	Proteins	Metabolites and Lipids		
1	✓	✓	✓	✓	A total of 203 DE transcripts, 164 DE proteins, and 58 DE GWAS-derived orthologs were found to be associated with GO-enriched pathways, associated mostly with lipid metabolism; Astrocytes and microglia are independently enriched in the AD-metabolic transcriptome; Based on the pathway enrichment analysis, lipid signatures, consisting of 133 species, were extracted and validated on human and mice metabolomic datasets. Significant coherence among datasets was found with dysregulation in cholesterol and phospholipids related pathways.	[63]
2	-	✓	-	✓	Twenty genes related to SM/Cer pathway were demonstrated DE in AD samples; As a result of the metabolomics dataset analysis, sphingosine-1-phosphate metabolism was found to be a key pathway associated with AD progression.	[64]
3	-	-	✓	✓	Oleamide concentrations were found to have significant impact on both AD progression and MCI/AD classification models; Phospholipase A2, properdin, alpha-synuclein, and junctophilin-3 were found to be key proteins predicting MCI conversion to AD; EVs extracted from microglia showed higher concentrations of oleamide compared to unstimulated and stimulated mice microglia cultures.	[65]
4	-	✓	✓	✓	Four miRNA’s—miR-28-5p, miR-381-3p, miR-651-5p, and miR-188-5p—demonstrated upregulation in AD cases’ EV samples; In AD EV samples, downregulation was observed for PE (38:0/38:1), lysoPE 2:4, and NAPE (16:0/18:0/20:4) and free cholesterol was the only upregulated lipid; Four proteins demonstrated decreased expression in the AD group and twenty-three proteins were found to be upregulated.	[70]
5	-	✓	✓	-	Ninety-three genes demonstrated the same dysregulation on both the proteome and transcriptome levels; Proteome profile of large and small EV subpopulation demonstrated overlap to the protein content of AD brains; Inflammatory pathway-related proteins were upregulated in AD compared to in EV subpopulations.	[71]

## 5. Bone Pathologies and Research Models

Bone-related diseases are closely associated with aging and significantly contribute to a decline in bone health. Like other tissues in the body, bones undergo a continuous remodeling process, where old bone is resorbed and new bone is formed. During youth, bone formation outpaces bone resorption, resulting in peak bone mass, typically achieved by around age 30. However, as individuals age, the balance between bone resorption and formation shifts, leading to a gradual loss of bone density. Bone aging contributes to age-related bone disorders such as osteoporosis, osteoarthritis, osteopenia, rheumatoid arthritis (RA), and periodontitis [72].

As life expectancy increases, the prevalence of bone-related diseases poses a significant public health challenge due to the increased risk of fractures, disability, and reduced quality of life in the aging population. Inhibitory processes, which are essential for optimal brain function, undergo age-related changes that may account for behavioral deficits. In particular, the inability to effectively modulate corticospinal excitability, which refers to the level of activity and excitability of the neural pathways connecting the cerebral cortex to the motor neurons of the spinal cord, has been associated with a decrease in motor activity in elderly individuals [73]. Gamma(γ)-aminobutyric acid (GABA), the primary inhibitory neurotransmitter, plays an important role in this process. GABA concentrations were significantly lower in patients with osteoporosis in the age group of 60–80 years (*p* = 0.04), whereas no such relationship was observed in younger women (48–59 years) [74]. Furthermore, genetic variants in two functional receptors for *GABA—GABBR2* (rs35126377 and rs11789969) and *GLRA1* (rs56177246 and rs145077031)—as well as in the necessary enzyme for its synthesis, *GAD1* (rs150390985)—showed an association with overall bone mineral density (BMD) among the combined cohorts. Moreover, *GLRA1* was significantly associated with overall BMD in the group aged over 60 years, while no such relationship was observed in the younger age groups [74].

A study on periodontitis was also published, comparing patients with the disease (mean age 44 years) and healthy participants (mean age 25 years) [75]. The study conducted the metabolomic profiling of patient samples, while transcriptomic analysis was based on a previously published dataset (GSE16134, mean age of patients 39,9 years) [76]. This compilation identified 146 differential metabolites and RNA sequencing revealed 102 differential genes associated with immune processes. The integration analysis, which included differential genes and metabolites, identified 33 key genes involved in cytokine-related pathways. Among them, *PDGFD*, *NRTN*, and *IL2RG* were identified as potential biomarkers of periodontitis and are suggested to influence disease progression by regulating deoxyinosine within the purine metabolism and Adenosine Triphosphate Binding Cassette transporter pathways [75].

Multiomics studies of age-related diseases associated with bone and joint tissues currently encompass only 23 articles. Most studies focus on the genome and transcriptome, with fewer addressing the metabolome and lipidome. For effective analysis, long-term studies of tissues from young to old age are necessary. Such studies not only require significant resources but also long-term access to tissue from healthy volunteers. Additionally, observing a group of individuals throughout their lives requires multiple generations of researchers and faces issues related to constantly changing methods and technologies for sample collection and analysis. Researchers are tasked with creating a model for studying aging. Key findings from some of these articles are summarized in Table 4.

A popular model for studying age-related diseases is animals, particularly mice. In a study by Li et al., a single-cell multiomics profiling approach was used to analyze bone marrow samples from mice of different ages (1, 6, and 20 months) to gain a comprehensive understanding of cellular changes over time [77]. As previously mentioned, genomic instability contributes to aging and telomeres, which play a crucial role in maintaining genomic stability, can be elongated by telomerase. To investigate the mechanisms underlying bone aging, the authors used telomerase-deficient mice (Terc^−/−^). These mice showed a significant increase in osteoclast (OC) differentiation compared to wild-type mice. Notably, 6-month-old Terc^−/−^ mice exhibited more pronounced signs of bone aging than 20-month-old wild-type mice. Telomerase dysfunction was associated with accelerated aging and osteoporosis, as well as changes in BMM and OC differentiation. Additionally, critical transcription factors (*Klf4, Cebpd, Irf8*, and *Sox4*) regulating osteoclast differentiation were identified and their knockdown disrupted this process [77].

Another commonly used model is cell lines, which, as previously shown, age at a rate approximately 40–140 times faster than in vivo (i.e., in the human body). Thus, 200–300 days in vitro corresponds to several decades of human life [78]. In an article by Sturm et al., a strategy was proposed for culturing fibroblasts found in connective tissue, which are responsible for producing collagen, elastin, and other components important for wound healing and tissue regeneration. The study profiled fibroblast cells taken from 6 healthy donors and 3 donors with *SURF1* gene mutations that disrupt the assembly and function of mitochondrial respiratory complex IV, leading to early death in affected patients [79]. Cells were cultured for 270 days. This multiomics dataset of cellular lifespan analysis includes longitudinal data across 13 major biological parameters, including cytological measures (growth rate and cell size), cellular bioenergetics (respiratory capacity and total energy consumption), transcriptomics (bulk RNA-seq), DNA methylation (EPIC array), whole genome sequencing (WGS), secreted factors (cytokines and cell-free DNA), and others [80]. All results have been made publicly available and can be reanalyzed, as well as visualized on the website [81].

## 6. Cancer

**Figure 1:**
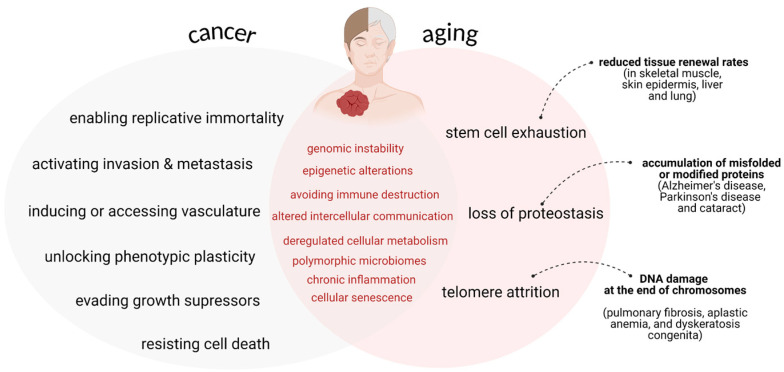
Cross-section of major “hallmarks” regarding cancer vs. aging.

Senescence has a proven role in the initiation and progression of cancer [82]. The primitive idea that somatic mutations drive tumorigenesis is commonly seen as the reason why most cancers develop in old age: as somatic mutations accumulate over time, the chance of reaching the oncogenic mutation threshold necessary for cancer initiation increases with age [83]. While this explanation is valid, it oversimplifies a much more complex process. It is well known that aging and cancer are both time-driven processes that share common “hallmark” mechanisms, namely genomic instability, epigenetic alterations, and chronic inflammation [84] (Figure 1). However, the idea of cancer being a result of accumulated somatic mutations is challenged by striking examples of long-lived species, such as bats and naked mole rats, as well as large organisms like elephants and whales [85,86].

The age-related paradox of oncological diseases is escalated by the fact that cancer differs from other age-related diseases because it involves a gain of function rather than a loss, allowing cells to proliferate uncontrollably, migrate, establish themselves in abnormal locations, resist apoptosis, and escape immune system detection [87]. Systematic text mining of scientific publications, electronic health records, and genome-wide association studies (GWAS) have indicated that aging remains the primary cancer risk factor: cancer frequency increased with age, peaking at 85 years [82,88]. Interestingly, in long-livers who surpass 90 years of age, cancer incidence and mortality show a significant decline. By the age of 100, cancer accounts for less than 5% of overall morbidity and mortality. This contrasts with the sharp rise in respiratory, infectious, and neurodegenerative diseases in elderly patients.

A null hypothesis has been proposed to explain the shift in the relationship between cancer and aging. It suggests that certain aspects of aging, such as telomere shortening and stem cell exhaustion, inhibit tumor formation. Other cancer hallmarks, e.g., impaired macroautophagy and cellular senescence, may have ambivalent effects, promoting and suppressing cancer. This creates a delicate balance between the mechanisms that drive and prevent cancer in an age-dependent manner [84].

Cancer is often referred to as a disease of aging; however, the mechanisms of how age affects cancer’s clinical behavior are still not well-known [89]. As could be expected from the complex interplay between tumorigenic molecular hallmarks and their antagonists, the task of understanding the age–cancer orchestra is far from simple [90].

Numerous attempts have been made to investigate the specific characteristics of cancer that emerge with age, but most of these efforts are limited by single omics [91]. Among all age-associated pathologies, cancer perhaps presents the most significant challenge for systematic multiomics analysis, which aims to differentiate between molecular causes and consequences.

This section outlines the local advances made in the systematic profiling of gero-cancers, with a focus on several specific cancer types. In the context of aging, we examined hormone-dependent breast cancer; thyroid cancer, where patient age serves as a diagnostic criterion; brain cancer, whose severity demands special attention; and lung cancer, which is strongly associated with external environmental factors (Table 5). Furthermore, we analyzed several key examples of pan-cancer studies conducted using a multiomics approach. It is important to include a disclaimer: studies that provide multiomic landscapes of specific cancer types do not typically prioritize the search for hidden connections between cancer pathogenesis and aging. However, the average age of patients in the studies we reference in this digest allows the multiomics findings to be interpreted in the context of cancer and aging.

### 6.1. TCGA-Wide Studies: Trying to Seize the Unseizable

Perhaps the most comprehensive study on cancer and aging to date is the recent integration of data from The Cancer Genome Atlas (TCGA) by Chatsirisupachai et al. [92]. The article offers a patchwork of cancer data, capturing both large-scale events and point mutations. This study focused on a systematic analysis of age-related differences in genomic instability (GI), loss of heterozygosity (LOH), whole-genome duplication (WGD), somatic copy-number alterations, point mutations, patterns of gene expression, and DNA methylation across 33 cancer types from over 9000 patients aged between 39 and 73 years. The authors identified several age-related pan-cancer and cancer-specific alterations. The highest rates of age-associated GI were observed in low-grade glioma, ovarian cancer, endometrial cancer, and sarcoma. Tumors from older patients tended to exhibit more unstable genomes and higher LOH levels in several cancer types. The “champions” of LOH were again low-grade glioma, endometrial cancer, and prostate adenocarcinoma. In contrast, lung adenocarcinoma, esophageal cancer, and liver cancer demonstrated a negative correlation between LOH and age. This inverse correlation may be influenced by factors such as differences in the distribution of age, smoking status, race, tumor grade, or other unexplained variables. The pan-cancer analysis revealed a slight increase in the likelihood of WGD with age. In cancer-specific analyses, a significant positive association was found in ovarian and endometrial cancers, indicating that tumors from older patients are more likely to have undergone genome doubling. The strongest association between age and increased GI, LOH, and WGD was observed in endometrial cancer, highlighting potential age-related disparities in the molecular landscape of this cancer type.

Another notable pan-cancer study analyzed the nuclear and mitochondrial somatic mutational landscapes at both the genomic and transcriptomic levels in the context of aging [89]. Drawing on data from over 20,000 tumors across 35 cancer types from TCGA, PCAWG, and GENIE datasets, the authors were able to assess how age dynamically influences the mutational burden within tumors (0.077 mutations per megabase per year). Specific mutational signatures were found to be associated with age, reflecting variations in exogenous and endogenous oncogenic processes. For example, tobacco-related mutational signatures were more prominent in lung tumors from younger patients, whereas DNA damage repair signatures were more active in tumors from older individuals. Notably, cancer-driver genes such as *CDKN2A* and *CREBBP* exhibited age-related mutation frequencies, with these alterations impacting the transcriptome and serving as predictors of clinical outcomes. These effects were particularly pronounced in brain cancers, where age-dependent alterations, such as *SUFU* loss and *ATRX* mutation, emerged as prognostic biomarkers. Alongside the findings of Chatsirisupachai et al. [92], this study underscored that characteristics of the tumor host play a pivotal role in shaping the tumor’s molecular profile, with some of these factors leading to age-specific transcriptomic and clinical outcomes.

### 6.2. Multifaced Breast Cancer in Your 30s and Your 70s

Significant progress has already been made in studying the age-specific characteristics of breast cancer [101]. It is well-established that the incidence of breast cancer rises sharply with age, particularly after 50 years. Breast cancer tends to be more common in older patients, while younger individuals are more likely to present with aggressive phenotypes and face considerably poorer prognosis [102].

To date, age-dependent frequencies of estrogen receptor-positive (ER+) and negative (ER−) breast cancer types have been identified and tests that detect predictive mutations (e.g., *BRCA1* [103]) have been developed and implemented in clinical practice. Among the many studies dedicated to the molecular profiles of breast cancer, one particularly noteworthy large-scale multiomics study compared gene expression in over 3000 breast cancer transcriptomes with epithelial protein expression in more than 5500 tumorous and normal breast tissues [93]. This study convincingly demonstrated that the predominant age-associated gene expression patterns in breast cancer are likely driven by gradual changes in age-related estrogen signaling, with smaller contributions from non-estrogen-sensitive genes. At the transcriptomic level, aberrations in the expression of *EZH2* (which negatively correlated with age) and *H3K27me3* (which positively correlated with age) were identified. While somewhat limited in scope due to their epithelial nature, the proteomic data from this study also revealed age-related prognostic significance for EZH2, H3K27me3, FOXA1, and BCL2.

Interestingly, breast cancer in the context of aging stands out among other cancer types, as estrogen signaling does not play a significant role in most cancers. While the pathogenesis of prostate cancer is closely linked to androgen signaling, the prostate cancer transcriptome has not revealed significant numbers of age-correlated transcripts [104].

### 6.3. Thyroid Cancer: When Age Is Staging Criterion

Thyroid cancer stands as the only type of cancer where patient age serves as a critical factor in both prognosis and staging [105]. Despite the clear-cut role of age in clinical decision-making, the molecular underpinnings of thyroid cancer development as a function of age remain incompletely understood. Age thresholds for staging have evolved without molecular justification; for instance, the American Joint Committee on Cancer (AJCC) set the age cutoff at 45 years in its 7th edition staging system, later adjusting this threshold to 55 years in the 8th edition. This shift has been supported by a recent multiomics study that integrated transcriptomic data, somatic mutation profiling, and DNA methylation analysis derived from the TCGA dataset [94]. The study uncovered age-related, DNA methylation-driven transcriptional regulation and identified specific somatic mutations, such as TTN and EIF1AX, as being particularly relevant to aging in thyroid cancer. Additionally, a predictive model based on three age-related genes (*PTK2B*, *E2F1*, and *GHR*) demonstrated promising accuracy in forecasting patient outcomes.

Shortly before that, Ruiz et al. conducted an integrative molecular analysis of over 400 thyroid tumor samples across varying ages [106]. Their findings revealed age-related molecular signatures with significant prognostic value for metastasis and survival outcomes. Central to their analysis was the *BRAF* mutation, particularly its role in the MAPK signaling pathway, which is critical for cell cycle regulation and survival [95]. Notably, the study demonstrated that aging, independent of the BRAFV600E mutation, is associated with a pronounced accumulation of molecular markers linked to aggressive tumor phenotypes and poor survival, particularly in patients aged 55 years and older. In addition, the study identified chromosomal alterations at loci 1p and 1q as key contributors to the increased tumor aggressiveness seen with age. Other hallmarks of aging thyroid tumors included reduced infiltration of CD8+ T cells and follicular helper T cells, along with dysregulated proteostasis, cellular senescence, and aberrant ERK1/2 signaling. A panel of 23 genes, including those involved in cell division (*CENPF*, *ERCC6L*, *MELK*, and *NEK2*), was defined as critical markers of both aging and tumor progression. These genes were instrumental in stratifying patients into aggressive subgroups, characterized by unique phenotypic and molecular profiles.

### 6.4. Lung Cancer: Nurture over Nature?

Traditionally, cancer pathogenesis research has focused on “nature,” prioritizing the search for key triggers among genetically driven determinants. In the case of lung cancer, however, the influence of “nurture” is evident, with smoking playing a significant role—its duration generally correlating with aging. While tobacco smoking is widely recognized as the primary risk factor for lung cancer, up to 25% of lung cancer cases occur in never-smokers [107]. A genomic study of lung cancer in never-smokers, conducted by the National Cancer Institute, identified three distinct molecular subtypes of the disease [108]. The study revealed that the majority of mutations in tumors from these patients were caused by natural, endogenous processes within the body, such as faulty DNA repair and oxidative stress, rather than by tobacco exposure. This research also highlighted that lung cancer in never-smokers (LCINS) occurs more frequently in women and at younger ages compared to smokers. The researchers identified three novel musical subtypes of LCISN based on the number of genomic changes (or noise) in the tumors. The dominant “piano” subtype, with the lowest mutational burden, grows slowly and is linked to progenitor cell activation. “Piano” features somatic *UBA1* mutations, germline AR variants, and stem cell-like properties, including high intratumor heterogeneity, long telomeres, and frequent *KRAS* mutations, as suggested by the occurrence of cancer drivers’ progenitor cells many years before tumor diagnosis. The “mezzo-forte” subtype has specific chromosomal changes and mutations in the *EGFR* gene, leading to faster tumor growth. The “forte” subtype, exhibiting whole-genome doubling, is characterized by rapid tumor progression, similar to cancers seen in smokers. Interestingly, no strong tobacco smoking signatures were detected in LCINS, even in cases with exposure to secondhand tobacco smoke. Genes within the receptor tyrosine kinase–Ras pathway had distinct impacts on survival; five genomic alterations independently doubled mortality. These findings suggest that different subtypes of lung cancer in never-smokers may require tailored approaches for prevention and treatment. The slow-growing “piano” subtype may offer a window for early detection, while the other subtypes could potentially benefit from targeted therapies.

Lung cancer heritability is estimated to be as high as 20% [109,110] and genome-wide association studies (GWAS) have identified over 50 risk loci to date. Many of these loci are specific to subgroups defined by histological subtype, ancestry, and, of course, smoking status, pointing to diverse mechanisms underlying lung cancer susceptibility. This dual behavior of lung cancer, alongside its high mortality, has driven numerous studies aimed at unraveling the molecular mechanisms of its pathogenesis.

A recent multiomics study focused on identifying effective biomarkers for improving prognosis and optimizing treatment in non-small cell lung cancer (NSCLC) patients [96]. The study used a seven-gene signature (*ABCC4*, *CCL19*, *CD27*, *DAG1*, *FUT7*, *SLC39A8*, and *ZNF71* [111,112]) to accurately predict recurrence and metastasis risk across 1500 early-stage NSCLC patients, covering all histological subtypes. Expanding their findings to the proteomic level, the authors highlighted ZNF71 as a key marker. When combined with dendritic cell activity, ZNF71 helps further stratify NSCLC into distinct prognostic groups. Overexpression of ZNF71 was linked to the reduced activity of intrinsic and innate immune system components, including dsRNA and dsDNA sensors, supporting the hypothesis that ZNF71 suppresses transcription of genomic transposable elements. Through computational network analysis, the team revealed multiomics interactions between ZNF71 and intracellular immune response genes, identifying genes with pan-sensitivity or resistance to 21 NCCN-recommended NSCLC therapies. This led to the development of potential therapeutic strategies aimed at enhancing treatment efficacy, inhibiting tumor proliferation, and reversing epithelial-mesenchymal transition.

In a recent study conducted by Wang et al. [97], a simplified model for predicting the tumor mutational burden (TMB) in patients with lung adenocarcinoma (LUAD) was developed using data from the TCGA database. Traditionally, TMB is calculated through whole-exome sequencing, which complicates its application in clinical practice. The researchers analyzed 610 differentially expressed genes, 50 differentially expressed microRNAs, and 58 differentially methylated CpG sites between high- and low-TMB groups. Based on these findings, four prognostic signatures were created, along with a predictive model for TMB utilizing machine learning methods that integrate expression or methylation profiles of 7 genes, 7 microRNAs, and 6 CpG sites [97].

Another related paper [98] conducted a comprehensive investigation into the impact of Intratumor Heterogeneity (ITH) on the effectiveness of bispecific antibody (bsAb) immunotherapy in patients with advanced NSCLC. Using advanced methodologies like Digital Spatial Profiling (DSP), NGS, and the nCounter platform, researchers analyzed transcriptomic and proteomic data from over 100 Regions of Interest. By considering ITH, they constructed protein signatures to predict immunotherapy outcomes and identified efficacy-related biomarkers from both tumor and stromal regions. Notably, stromal areas exhibited richer genetic information, enhancing predictive capabilities. Key protein markers linked to tumor phenotypes included epithelial cell adhesion molecule, HER2, and Ki-67, while immune cell markers (CD45) showed significant elevation in stromal zones. This comprehensive approach, leveraging DSP, addressed the limitations of traditional bulk sequencing and identified 18 protein candidates as potential biomarkers for predicting the efficacy of bsAb immunotherapy targeting PD-L1 and CTLA-4.

### 6.5. Glioblastoma Case

Glioblastoma (GBM) is the most common and deadly form of brain tumor, with a median survival time of only 15 months—a statistic that has seen little improvement over the past few decades. A deeper understanding of the molecular basis of GBM is urgently needed to improve therapeutic outcomes and survival rates. In a collection of over 500 untreated glioblastoma samples (median donor age = 59.6 years), a comprehensive landscape of genomic, transcriptomic, methylomic, and proteomic alterations was established, alongside the identification of molecular subtypes and affected pathways [99]. This study identified distinct molecular subtypes and key affected pathways. Several significantly mutated genes (such as *PTEN, TP53, EGFR, PIK3CA, PIK3R1, NF1, RB1, IDH1, LZTR1*, and *PDGFRA*) were detected, along with complex rearrangements of signature receptors and a wide array of altered transcripts. Mutations in the TERT promoter were associated with increased mRNA expression, suggesting a role in telomerase reactivation. Correlational analyses highlighted that the survival benefit of the proneural subtype was linked to the G-CIMP phenotype [113] and *MGMT* DNA methylation emerged as a predictive biomarker for treatment response in the classical GBM subtype. Proteomic data were the cherry on top of this multiomics study and allowed to divide the cohort under study into clearly-defined subtypes.

Another notable study [100] on glioblastoma, whose incidence peaks between the ages of 65 and 75, found 11 genes (*UBC, HDAC1, CTNNB1, TRIM28, CSNK2A1, RBBP4, TP53, APP, DAB1, PINK1*, and *RELN*), five miRNAs (hsa-mir-221-3p, hsa-mir-30a-5p, hsa-mir-15a-5p, hsa-mir-130a-3p, and hsa-let-7b-5p), six metabolites (including N6-acetyl-L-lysine, cholesterol, and formate), and 15 distinct signaling pathways that are essential for the development of glioblastoma. The top gene, miRNA, and metabolite signatures identified in this study may be used to develop early diagnostic procedures and construct individualized therapeutic approaches to glioblastoma.

## 7. Is There a Better Way for Multiomics Data Processing?

The integration of multiomics data remains a challenging task due to the high variability within a single dataset and the combined influence of multiple factors, which complicates the identification of biologically significant and independent molecules [114]. A potential solution lies in the standardization of sample preparation and data analysis methods, which can minimize technical variability between studies and improve result reproducibility [115]. Furthermore, the integration of multiomics data into long-term population studies with regular data collection at different life stages can facilitate the tracking of aging dynamics and account for factor changes over time. This approach has the potential to significantly enhance the understanding of the biological mechanisms of aging and provide a foundation for the development of targeted interventions.

Besides the actual data complexity peculiar to single-omics studies, a multiomics strategy assumes the usage of intelligent workflows for integration of data from multiple layers of biological information. Despite the critical role of this step, many published studies disregard the actual integration step, processing data separately, possibly missing important level-to-level patterns and connections. Nonetheless, the availability of such datasets makes it possible to provide integration or even process them with other data.

Talking about actual data integration strategies, there are three main groups of methods: conceptual, statistical, and network-based integration [116]. The first group of methods includes multi-level data incorporation based on the common genes, pathways, or even diseases which are associated with features derived from the different levels. Popular key pillars for such integration include Gene Orthologies (GO), pathways (Reactome- or KEGG-based) and Disease Orthologies (DO). However, it should be noted that the conceptual approach utilizes data based on the current state of knowledge on biological processes, which results in low efficiency in terms of uncovering relationships and patterns, so it is common to see this branch of tools used in combination with other integration strategies. The utilization of the conceptual approach is common for aging-related disease studies, mostly in the context of GO [117,118] and pathway [119] analysis.

As an orthogonal approach, we have the statistical integration group of methods which are based on the combination and comparison of quantitative data from analyzed datasets using various statistical tools. Compared to the conceptual approach, utilizing statistics in most cases “homogenizes” data, allowing to discover hidden connections between the studied levels. We can broadly categorize those methods into several groups based on the statistical frameworks they use: regression-based approaches, correlation-based methods, latent variable models, machine learning-based methods, etc. Usage of sPLS-DA [120] and multiomics factor analysis [121] was described above in the context of AD related studies.

The last integration strategy stands on the overlap of the conceptual and statistical approaches and utilizes network-based representation of data. Based on the actual tools used, nodes in such networks will represent biological entities such as genes, proteins, metabolites, or pathways, with edges describing the relationships between the nodes. A good example of network-based approach utilization was provided by Yang et al. [122]. Researchers used a similarity network fusion (SNF) method for processing DNA methylation, transcriptomics, proteomics, H3K9 acetylation, and metabolomics datasets simultaneously in order to detect molecular subtypes associated with cognitive decline.

In the context of the analysis of several cohorts’ datasets simultaneously, database-based tools are very useful. A tool entitled “Network OMics Ageing DataBase”m (NOMA-DB) [123] is a framework designed to analyze gene expression data related to aging. It aggregates gene expression data from the Genotype-Tissue Expression database and combines it with information from protein–protein interaction databases. This integration enables exploring gene expression variety across different tissues and protein interactions within aging-related pathways.

For the effective analysis of multiomics data and integration of various biological layers, the use of specialized tools and approaches is required. These tools enable the processing, analysis, and visualization of large volumes of data, facilitating the identification of hidden patterns and interactions between different omics layers (Table 6).

## 8. Conclusions

Aging represents a collection of biological processes occurring at the cellular and organismal levels, accompanied by a gradual deterioration of physiological functions, making age a significant risk factor for the development of various diseases. In reviewing the literature, we aimed to identify pathways, genes, proteins, or metabolites associated with cellular aging. However, we encountered several limitations. As previously noted, there is no clear definition of “healthy” aging. Most studies on healthy aging lack strict control over the presence of pathologies. Absolute health is unlikely, yet the boundaries defining what is considered an acceptable level of health remain undefined.

There is also a limited number of studies that truly compare diseases in old age with those in youth, making it difficult to distinguish pathological signs from general signs of physiological wear. Thus, currently, there are no standards or clear criteria to separate healthy aging from pathological aging, which complicates the development of an objective model of “healthy” aging. Most studies focus on models based on cell lines subjected to artificial aging by prolonged culture or treatments, such as hydrogen peroxide, or on animal models like mice.

An interesting alternative for studying aging processes could be progeria—a syndrome that resembles certain physiological aspects of aging. Progeria could serve as a useful model for studying conditions such as CVD, calcification, atherosclerosis, adipogenesis, and osteogenesis. However, it is less suitable for studying oncological processes despite the high frequency of DNA damage in cell lines like fibroblasts. Paradoxically, progeria may actually inhibit cancer development: the accumulation of the pathological protein prelamin A could prevent cancer cell invasion and carcinoma infiltration [133].

Progeria is also not an optimal model for studying neurodegenerative diseases and immune deficiencies. The low level of progerin in the brain and the effect of microRNA miR-9, which suppresses lamin A synthesis, make it ineffective for modeling processes in the central nervous system.

**Figure 2:**
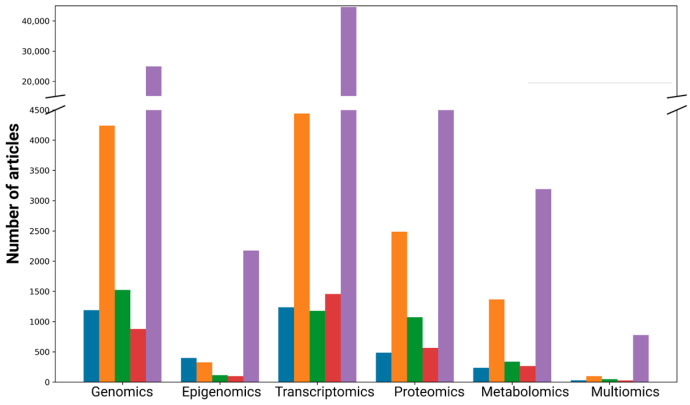
The distribution of PubMed articles related to omics studies across various disease categories. Each color represents a different category: blue—aging, orange—CVD, green—neurodegenerative diseases, red—bone-related diseases, and purple—cancer.

In our literature review, we expected to find a significant number of studies integrating multiple omics layers to comprehensively study aging. However, the volume of experimental research dedicated to aging is comparable to studies on neurodegenerative and bone diseases and is significantly lower than research on cardiovascular diseases, which are studied three times as frequently (Figure 2). Even in oncology, which leads in the number of age-related pathology studies, the majority of work (around 70,000 studies) is done at the genome and transcriptome levels. Only 706 articles include multiomics profiling and the multiomics approach is used even less frequently in aging research—in fewer than 200 studies. Although it may seem that multiomics studies for cancer are abundant, a deeper analysis reveals that the integration of omics layers is rare. Most often, different omics levels are considered in isolation and conclusions are drawn from each separately, without attempts to connect the data to create a cohesive picture.

To date, most information on age-related processes has been derived from genomic and transcriptomic data, which serves as a valuable foundation for deciphering the mechanisms of aging and associated pathologies. A comprehensive understanding of aging is only achievable through the integrated analysis of all levels of genome implementation, allowing for the identification of specific and general biological scenarios, their interconnections, and their regulatory mechanisms.

## Figures and Tables

**Table 4:** Summary of multiomics research discoveries on bone-related diseases.

#	Omics Layers					Bullet Points	Ref.
	DNA/Methylated DNA	RNA	Proteins	Metabolites and Lipids			
1	✓	-	-	✓	-	GABA concentrations were significantly lower in osteoporosis patients aged 60–80 years (*p* = 0.04), but this relationship was not observed in younger women (48–59 years); Genetic variants in functional receptors for GABA, including GABBR2 (rs35126377 and rs11789969) and GLRA1 (rs56177246 and rs145077031), as well as in the synthesis enzyme GAD1 (rs150390985), are associated with overall bone mineral density (BMD) across combined cohorts; The GLRA1 genetic variant showed a significant association with overall BMD, specifically in individuals aged over 60 years, with no such association found in younger groups.	[74]
2	✓	✓	-	-	-	Telomerase-deficient mice (Terc^−/−^) demonstrated increased osteoclast (OC) differentiation compared to wild-type mice; Bone aging was more pronounced in 6-month-old Terc^−/−^ mice than in 20-month-old wild-type mice, highlighting the role of telomerase dysfunction in accelerated aging and osteoporosis; Alterations in bone marrow microenvironment (BMM) and OC differentiation were observed in Terc^−/−^ mice; Key transcription factors regulating osteoclast differentiation, including Klf4, Cebpd, Irf8, and Sox4, were identified and their knockdown disrupted this process.	[77]

**Table 5:** Summary of multiomics research discoveries on cancer.

#	Omics Layers					Bullet Points	Ref.
	DNA/Methylated DNA	RNA	Proteins	Metabolites and Lipids	Microbiota		
Pan-cancer studies							
1	✓	✓	-	-	-	Tumors in older patients exhibit increased genomic instability, somatic copy-number alterations, and mutations, with the most pronounced age-related differences seen in gliomas and endometrial cancer; Age-related DNA methylation affects key cancer genes.	[92]
2	✓	✓	-	-	-	Age affects both the number and timing of tumor mutations. Mutational signatures reveal age-specific oncogenic processes (e.g., tobacco has a greater impact in younger patients and DNA repair activity is higher in older ones). Key cancer genes (*CDKN2A* and *CREBBP*) show age-related mutation frequencies, altering the transcriptome, particularly in brain cancers, where *SUFU* loss and *ATRX* mutations serve as age-dependent prognostic biomarkers.	[89]
Breast cancer							
3	-	✓	✓	-	-	Most age-correlated genes, such as *EZH2* (−correlation) and *H3K27me3* (+correlation), are influenced by age-dependent estrogen signaling; An age-stratified analysis of breast cancer outcomes using EZH2, H3K27me3, FOXA1, and BCL2 proteins demonstrated their prognostic significance; Age-related gene expression may play a critical role in evaluating *ER*, *EZH2*, and *H3K27me3*.	[93]
Thyroid cancer							
4	✓	✓	-	-	-	Age is an independent risk factor for overall and progression-free survival in differentiated thyroid cancer, with 45 years serving as a key therapeutic cutoff; Aging influences DNA methylation-driven transcriptional regulation and distinct somatic mutation patterns, including *TTN* and *EIF1AX*, have been identified in young and older patients; A prognostic model was developed based on three age-related genes: *PTK2B*, *E2F1*, and *GHR*.	[94]
5	✓	✓	-	-	-	Aging, independent of the BRAFV600E mutation, increases markers of tumor aggressiveness; Chromosomal alterations at 1p/1q, reduced infiltration of CD8+ T and follicular helper T cells, dysregulated proteostasis and senescence, and activation of the ERK1/2 signaling cascade are key drivers of thyroid cancer progression in older, but not younger, patients; *CENPF*, *ERCC6L*, *MELK*, *NEK2*, and 19 other genes serve as age-dependent aggressiveness markers and predict metastasis stage and survival.	[95]
Lung cancer							
6	-	✓	✓	-	-	ZNF71 expression, combined with dendritic cell activity, defines lung cancer subgroups with distinct survival outcomes; Overexpression of ZNF71 suppresses key components of the intrinsic and innate immune systems, including dsRNA and dsDNA sensors; Multiomics analysis linked ZNF71 to tumorigenesis, proliferation, and survival, integrating clinical data with CRISPR-Cas9 and RNAi screening; Potential targeted therapies identified include MEK1/2 inhibitors (PD-198306 and U-0126), VEGFR inhibitor (ZM-306416), and IGF-1R inhibitor (PQ-401), which may enhance immune responses in treatment.	[96]
7	✓	✓	-	-	-	Machine learning analysis of differentially expressed genes, miRNAs, and methylated CpG between high and low tumor mutation burden groups resulted in four predictive signatures for lung adenocarcinoma.	[97]
8	-	✓	✓	-	-	Digital spatial profiling of tumor and stromal regions at the proteomic and transcriptomic levels revealed that the stromal-derived signature had superior predictive power for immunotherapy response.	[98]
Glioblastoma							
9	✓	✓	✓	-	-	Significantly mutated genes (*PTEN, TP53, EGFR, PIK3CA, PIK3R1, NF1, RB1, IDH1, LZTR1,* and *PDGFRA*) were identified as potential glioblastoma markers, alongside complex receptor rearrangements and altered transcripts; The survival advantage of the proneural subtype is linked to the G-CIMP phenotype, while *MGMT* DNA methylation may predict treatment response only in the classical glioblastoma subtype.	[99]
10	✓	✓	✓	✓	-	Eleven genes (*UBC, HDAC1, CTNNB1, TRIM28, CSNK2A1, RBBP4, TP53, APP, DAB1, PINK1,* and *RELN*), five miRNAs, six metabolites, and fifteen distinct signaling pathways play an indispensable role in glioblastoma.	[100]

**Table 6:** Tools for multiomics data integration.

#	Tool	Description	Ref.
1	Multiomics factor analysis (MOFA)	Latent factor model Uses Bayesian factor analysis to identify shared and specific sources of variability across datasets. Identification of latent factors linking omics layers (e.g., genomics, proteomics, and metabolomics).	[124]
2	DIABLO (Data Integration Analysis for Biomarker discovery using Latent Variable approaches for Omics studies)	Sparse partial least squares regression (sPLS-DA). Integrates multiomics datasets using supervised multivariate analysis. Performs feature selection across multiple data layers.	[125]
3	iClusterplus	Performs Gaussian latent variable modeling to integrate heterogeneous datasets. Identifies clusters or subtypes by summarizing multiomics datasets.	[126]
4	MINT (Multiomics INTegration)	Extends sPLS to integrate datasets from multiple studies or cohorts. Focuses on classification tasks using supervised learning	[127]
5	Multiomics Module Analysis (MOMA)	Module-based integration. Groups omics features into co-regulated modules using statistical associations.	[128]
6	RGCCA (Regularized Generalized Canonical Correlation Analysis)	Regularized multivariate analysis. Extends canonical correlation analysis with regularization to handle high-dimensional data.	[129]
7	Similarity Network Fusion	Combines similarity networks from multiple omics datasets into a unified consensus network.	[130]
8	BioUML	This is a web-based platform designed for data integration and analysis in systems biology. It enables visual modeling and the creation of hierarchical biological models, facilitating the development of complex modular frameworks.	[131]
9	ReactomeFI	Combines omics data with Reactome pathways.	[132]
10	NOMA-DB	Framework for ageing studies based on an extension of the GTEx database that enable easy querying at the sex/age level and network-based analysis.	[123]
